# Insights Into Hereditary Alpha‐Tryptasemia From a Genome‐Wide Association Study in Hymenoptera Venom Anaphylaxis

**DOI:** 10.1111/cea.70211

**Published:** 2026-01-07

**Authors:** Teresa Blanco‐Ramos, Raquel Cruz, Irene Fernández‐Franco, Mª Ángeles Vargas, Ángel Carracedo, Arturo González‐Quintela, Carmen Vidal

**Affiliations:** ^1^ Department of Medicine, Faculty of Medicine Universidad de Santiago de Compostela (USC) Santiago de Compostela Spain; ^2^ Fundación Pública Galega de Medicina Xenómica Fundación Instituto de Investigación Sanitaria (FIDIS) Servicio Galego de Saúde (SERGAS) Santiago de Compostela Spain; ^3^ Genomics and Bioinformatics Group, Center for Research in Molecular Medicine and Chronic Diseases (CiMUS) Universidade de Santiago de Compostela (USC) Santiago de Compostela Spain; ^4^ Allergy Service Complejo Hospitalario Universitario de Santiago Santiago de Compostela Spain; ^5^ Centro de Investigación en Red de Enfermedades Raras (CIBERER) Universidade de Santiago de Compostela (USC) Santiago de Compostela Spain; ^6^ Internal Medicine Service Complejo Hospitalario Universitario de Santiago Santiago de Compostela Spain

**Keywords:** anaphylaxis, GWAS, hereditary alpha‐tryptasemia (HαT), hymenoptera, mast cell activation syndrome (MCAS), mast cell disorder, venom allergy

## Abstract

- The G allele in rs111911698 is strongly associated with BST ≥ 8 ng/mL and could serve as a genetic proxy for the detection of HαT in patients with Hymenoptera venom anaphylaxis.
- The G allele in rs111911698 is a highly sensitive and specific identifier of HαT, with high positive and negative likelihood ratios.

**Abbreviations**
BST: basal serum tryptase
GWAS: genome‐wide association study
HVA: hymenoptera venom allergy
HαT: hereditary alpha‐tryptasemia
VIT: venom immunotherapy

To the Editor,

Systemic reactions to Hymenoptera stings constitute one of the most prevalent and severe forms of anaphylaxis [1]. A high prevalence of up to 11.6% of elevated basal serum tryptase (BST) has been reported in this context among European populations [2]. Since 2016, hereditary alpha‐tryptasemia (HαT) has emerged as a plausible explanation for otherwise unexplained high BST levels [3]. HαT is an autosomal dominant trait caused by increased germline copy number of the *TPSAB1* gene, which encodes α‐tryptase [3]. The prevalence of this condition is estimated to range from 5% to 7.5% among the European [4] and US [5] populations. A serum tryptase cut‐off level of 8 ng/mL has been proposed as a means of suspecting HαT [6].

A genome‐wide association study (GWAS) in a cohort of patients undergoing venom immunotherapy (VIT) is presented to identify genetic variants that may be associated with tryptase levels.

This observational, prospective study recruited 281 patients (221, 78.6%, male, mean age [56.8 ± 14.8 years]) who attended the Allergy Department to receive VIT, between April and December 2021. The grading system for generalised hypersensitivity reactions proposed by Brown (mild [*n* = 73], moderate [*n* = 114], or severe [*n* = 94]) [7] was used. A more severe anaphylaxis was observed to be associated with increasing age (54 [39–66], 59 [45–68], 63 [53–68] years old; *p* = 0.011), and higher BST levels (4.6 [3.5–5.7], 4.7 [3.7–6.3], 5.4 [4.3–6.7] ng/mL; *p* = 0.002). A REMA score was calculated for all patients and 15 (5.4%) with ≥ 2 values were studied at the Haematology Service, ruling out mastocytosis after bone marrow biopsy and examination for c‐KIT p.D816V in peripheral blood leukocytes.

Prior to inclusion, all participants were informed about the nature and aims of the study, and consent was sought. The study was approved by the Institutional Research Ethics Committee (CEIm‐G) and adhered to the Declaration of Helsinki.

DNA was isolated from blood samples (collected with EDTA) using Chemagen's Magnetic‐Beads kit (Revvity). DNA concentration and integrity were determined using Nanodrop ND‐1000 (ThermoFisher Scientific). Samples were genotyped with the Axiom Spain Biobank Array (Thermo Fisher Scientific) at the Santiago de Compostela node of CeGen (Centro Nacional de Genotyping). PLINK 1.9 and custom R scripts were used to check the quality of genetic data (additional information file is available at Zenodo: https://doi.org/10.5281/zenodo.17914583). Imputation was performed using TOPMed r2 version (NHLBI Trans‐Omics for Precision Medicine).

Only thirty‐one patients presented a BST ≥ 8 ng/mL. These patients were further analysed for HαT‐associated *TPSAB1* gene by droplet digital PCR (ddPCR) (Bio‐Rad) and data acquisition and analysis was performed on the QX200 Droplet Reader (Bio‐Rad) using QuantaSoft Software (Bio‐Rad). To quantify the number of copies of the α and β tryptase coding alleles, the specific primers TPSAB—FWD 5′‐TCCTGACCTGGCACCTGC‐3′ and TPSAB—REV 5′‐GACTCTCAGGCTCACCTGCCA‐3′ and the specific probes for α: 5′‐CTGCAGCAAGCGGGTATCGTC‐3′ and for β: 5′‐CTGCAGCGAGTGGGCATCGT‐3′ (Applied Biosystems), both labelled with FAM [3]. The VIC‐labelled RNase P assay (Applied Biosystems) was used as a reference assay for copy number. HαT was defined as three or more copies of α‐tryptase whatever the β‐copies or two copies of α‐tryptase in the presence of 3β‐copies. These experiments were performed at the DNA Research Support Service‐Nucleus, University of Salamanca.

When GWAS was performed using the tryptase value dichotomously (< vs. ≥ 8 ng/mL) as the dependent variable in the logistic regression model, and using gender and age as covariates, a significant locus (*p* < 5 × 10^−8^) was found on chromosome 16p13, where the cluster of tryptase genes is located. The regional plot, depicting the significant region surrounding the lead SNP in *TPSG1* (rs111911698, located in an intron of *TPSG1*), identified four genes—*CACNA1H, TPSAB1, TPSG1* and *TPSD1*—highlighted by positional and eQTL mapping in FUMA (Figure 1).

**FIGURE 1:**
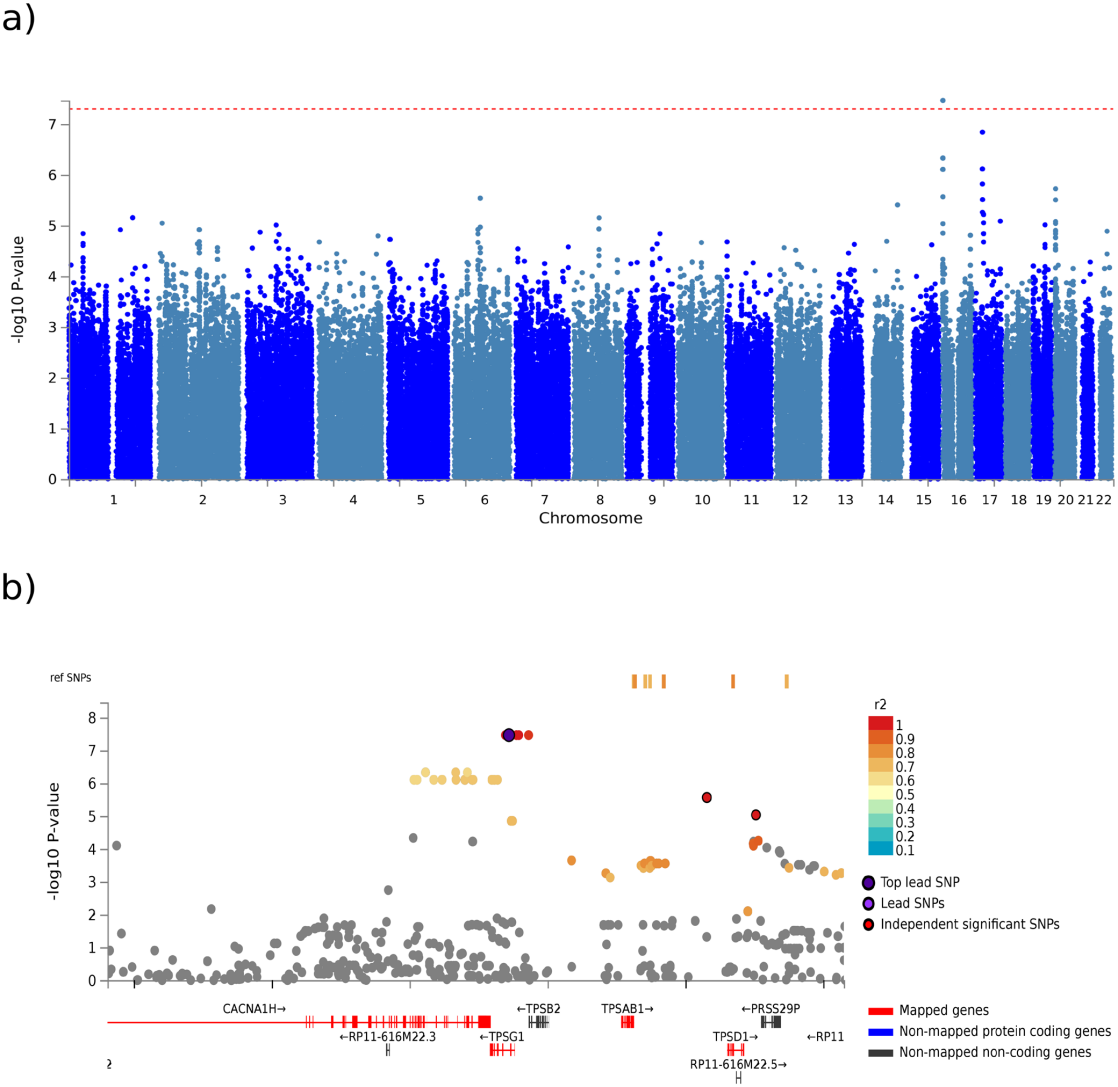
(a) Manhattan plot of the GWAS for risk of tryptase levels > 8 ng/mL (suggested threshold for HαT search, adjusting for covariates: gender, age). The results show the *y* axis, the 10,442,748 genetic markers (SNPs), and on the *x* axis, their genomic location on the 23 chromosomes. The blue transverse line marks the 5 × 10^−5^ significance point and the red transverse line marks the 5 × 10^−8^ significance point. There is one SNP that reach statistical significance located on chromosome 16. (b) Regional plot around TPSG1 SNP. The imputation results find several genes in the region surrounding SNP rs111911698, specifically CACNA1H, TPSAB1, TPSG1, and TPSD1.

Only 12/31 (38.7%) patients tested for HαT by ddPCR were confirmed among patients with BST ≥ 8 ng/mL. Notably, the lowest BST level among HαT carriers was 11 ng/mL, supporting the redefinition of the previously proposed threshold of 8 ng/mL to 11 ng/mL [8]. If such a threshold were adopted, 63.1% (12/19) of our patients would be classified as HαT carriers. The regional plot surrounding the lead SNP of *TPSG1* (rs111911698), detected in the GWAS analysis, highlighted the *CACNA1H* gene, which Lyons had already described as adjacent to the tryptase locus [9].

When analysing the subgroup of patients with BST ≥ 8 ng/mL, who had undergone α‐ and β‐tryptase copy number analysis, comparison with GWAS results revealed a high correlation between the two techniques. Specifically, the lead SNP on chromosome 16 (rs111911698) was selected for investigation, and its association with HαT was determined. The results revealed that 11 out of 12 patients diagnosed with HαT were found to be carriers of the G allele, in contrast to only 2 out of 19 individuals with BST ≥ 8 ng/mL but without HαT (*p* = 0.00001). The association remained significant when a logistic regression analysis was performed (using HαT status as the dependent variable) with adjustments for sex and age (*p* = 0.00078). Therefore, the lead SNP rs111911698 demonstrated a sensitivity of 92% [62%–100%], a specificity of 89% [67%–99%], a positive likelihood ratio of 8.71 [2.32–32.66], and a negative likelihood ratio of 0.09 [0.01–0.61] for the identification of HαT. These results suggest that the presence of the G allele in rs111911698 could be used as a proxy for the presence of HαT, giving an easier test to be performed to identify HαT, particularly in terms of workflow complexity, equipment requirements, and accessibility in standard laboratory settings.

## Author Contributions

Teresa Blanco‐Ramos, Irene Fernández‐Franco, and Mª Angeles Vargas collected the clinical information of the participants and conducted the literature review. Raquel Cruz and Arturo González‐Quintela performed the statistical analysis and contributed to the writing of the result section. Angel Carracedo supported the research concept. Carmen Vidal is the principal investigator of the study, developed the research concept and wrote the final version of the manuscript which was discussed, reviewed and approved by all authors.

## Funding

The study was supported by a grant from the FSEAIC (Fundación de la Sociedad Española de Alergología e Inmunología Clínica) 2019.

## Ethics Statement

The study was approved by the Regional Ethics Committee (code 2010–315).

## Consent

Written informed consent was obtained from all participants.

## Conflicts of Interest

The authors declare no conflicts of interest.

## Data Availability

The data that support the findings of this study are available on request from the corresponding author. The data are not publicly available due to privacy or ethical restrictions.
